# The effects of creatine supplementation on cognitive function in adults: a systematic review and meta-analysis

**DOI:** 10.3389/fnut.2024.1424972

**Published:** 2024-07-12

**Authors:** Chen Xu, Siyuan Bi, Wenxin Zhang, Lin Luo

**Affiliations:** School of Physical Education, Guizhou Normal University, Guiyang, China

**Keywords:** creatine, cognitive function, brain health, neuropsychological tests, randomized controlled trials

## Abstract

**Background:**

This study aimed to evaluate the effects of creatine monohydrate supplementation on cognitive function in adults and explore its potential role in preventing and delaying cognitive impairment-related diseases.

**Methods:**

Following the PRISMA 2020 guidelines, a systematic review with meta-analysis was conducted. Randomized controlled trials (RCTs) published between 1993 and 2024 were retrieved from PubMed, Scopus, and Web of Science databases. The study protocol was registered with PROSPERO (registration number: CRD42024533557). The impact of creatine supplementation on overall cognitive function, memory, executive function, attention, and information processing speed was assessed using standardized mean differences (SMD) and Hedge’s g with 95% confidence intervals (CI).

**Results:**

Sixteen RCTs involving 492 participants aged 20.8–76.4 years, including healthy individuals and patients with specific diseases, were selected. Creatine monohydrate was the form used in all included studies. Creatine supplementation showed significant positive effects on memory (SMD = 0.31, 95% CI: 0.18–0.44, Hedges’s *g* = 0.3003, 95% CI: 0.1778–0.4228) and attention time (SMD = −0.31, 95% CI: −0.58 to −0.03, Hedges’s *g* = −0.3004, 95% CI: −0.5719 to −0.0289), as well as significantly improving processing speed time (SMD = −0.51, 95% CI: −1.01 to −0.01, Hedges’s *g* = −0.4916, 95% CI: −0.7852 to −0.1980). However, no significant improvements were found on overall cognitive function or executive function. Subgroup analyses revealed that creatine supplementation was more beneficial in individuals with diseases, those aged 18–60 years, and females. No significant differences were found between short- (<4 weeks) and long-term (≥4 weeks) interventions for improving cognitive function. Low-to-moderate risk of bias was found, and no significant publication bias was detected. The GRADE assessment indicates that the certainty of evidence for memory function is moderate, suggesting a reasonable level of confidence in the positive effects of creatine on memory. However, the evidence for processing speed, overall cognitive function, executive function, and attention is of low certainty, indicating that further research is needed to confirm these potential benefits.

**Conclusion:**

Current evidence suggests that creatine monohydrate supplementation may confer beneficial effects on cognitive function in adults, particularly in the domains of memory, attention time, and information processing speed. Larger robust clinical trials are warranted to further validate these findings. Furthermore, future research should investigate the influence of different populations and intervention durations on the effects of creatine monohydrate supplementation, as well as elucidate the precise mechanisms underlying its potential cognitive-enhancing properties.

## 1 Introduction

Creatine, a nitrogenous organic acid naturally occurring in vertebrates, plays a critical role in the energy metabolism of brain cells (1). Synthesized primarily from arginine, glycine, and methionine, creatine is produced endogenously and obtained through dietary intake. After cellular uptake, creatine is converted into phosphocreatine (PCr), which is rapidly broken down via catalysis by creatine kinase (CK) to facilitate adenosine triphosphate (ATP) regeneration, thereby serving as a crucial element in energy transfer (2–5). While widely utilized for enhancing athletic performance and promoting overall health, creatine supplementation has garnered increasing interest for its potential cognitive benefits (6–10).

The global rise in aging populations has led to a concurrent increase in the prevalence of cognitive impairment among individuals aged 65 and older. These impairments encompass deficits in a range of cognitive functions, including attention, memory, executive function, language, and processing speed—all of which are essential for daily living and social interaction. As individuals age, these cognitive functions may experience significant decline, impacting judgment, decision-making, and the ability to perform everyday tasks (11–13). Age-related cognitive decline, particularly neurodegenerative diseases like Alzheimer’s disease (AD), has emerged as a major public health concern (14).

Although preliminary research has explored the potential of creatine supplementation in enhancing cognitive function (15), existing studies are often characterized by limited sample sizes and inconsistent findings, lacking a systematic synthesis. This study aims to systematically evaluate the effects of creatine supplementation on cognitive function in adults through meta-analysis, with a particular focus on memory, executive function, attention, and processing speed. This meta-analysis aims to provide comprehensive evidence regarding the effects of creatine supplementation on cognitive performance, identifying cognitive domains that may benefit most from this intervention. Furthermore, by analyzing the heterogeneity across studies, this research will offer insights to guide future investigations. The findings of this study are expected to provide a scientific foundation for developing non-pharmacological intervention strategies to mitigate cognitive decline, enhance quality of life, and potentially contribute to the prevention of cognitive-related diseases.

## 2 Methods

### 2.1 Protocol and registration

This systematic review and meta-analysis was conducted in accordance with the Preferred Reporting Items for Systematic Reviews and Meta-Analyses (PRISMA) 2020 guidelines. The study protocol was registered with PROSPERO (registration number: CRD42024533557) prior to the literature search.

### 2.2 Study design

A systematic review with meta-analysis was performed to comprehensively evaluate the effects of creatine supplementation on cognitive function in adults. This study particularly focused on the potential differences in cognitive performance among adult populations with different health conditions (including healthy and diseased populations) and demographic characteristics (age: over 60 years old and 18–60 years old; sex: male, female) under creatine supplementation.

### 2.3 Search strategy

This systematic review and meta-analysis investigated the effects of creatine supplementation on cognitive function in adults. The search strategy encompassed three electronic databases—PubMed, Scopus, and Web of Science (WOS)—spanning from January 1, 1993, to June 5, 2024. The search strategy adhered to the PICO principle (Table 1), as detailed below:

**Table 1:** PICO search strategy elements.

PICO element	Description	Search terms
P (Population)	Adults aged 18 years and older	adult*, adults, aged, aging, elderly*
I (Intervention)	Creatine supplementation	creatine, “creatine supplement*,” CrM, “creatine monohydrate”
C (Comparison)	Placebo or no intervention	placebo*, control*, “no intervention”
O (Outcome)	Cognitive function	cognition, “cognitive function*,” memory, “executive function*,” attention, “processing speed”

Search strategies were tailored to the specific syntax of each database, employing Boolean operators (AND, OR, NOT) to combine search terms effectively.

#### 2.3.1 PubMed

(“creatine”[Title/Abstract] OR “creatine supplement*”[Title/Abstract] OR CrM[Title/Abstract] OR “creatine monohydrate”[Title/Abstract]) AND (cognition[Title/Abstract] OR “cognitive function*”[Title/Abstract] OR memory[Title/Abstract] OR “executive function*”[Title/Abstract] OR attention[Title/Abstract] OR “processing speed”[Title/Abstract]) AND (adult*[Title/Abstract] OR adults[Title/Abstract] OR aged[Title/Abstract] OR aging[Title/Abstract] OR elderly*[Title/Abstract]) NOT (animals[Title/Abstract] OR children[Title/Abstract]).

#### 2.3.2 Scopus

TITLE-ABS-KEY (creatine OR “creatine supplement*” OR CrM OR “creatine monohydrate”) AND TITLE-ABS-KEY (cognition OR “cognitive function*” OR memory OR “executive function*” OR attention OR “processing speed”) AND TITLE-ABS-KEY (adult* OR adults OR aged OR aging OR elderly*) AND NOT TITLE-ABS-KEY (animal OR children).

#### 2.3.3 Web of science

TS = (creatine OR “creatine supplement*” OR CrM OR “creatine monohydrate”) AND TS = (cognition OR “cognitive function*” OR memory OR “executive function*” OR attention OR “processing speed”) AND TS = (adult* OR adults OR aged OR aging OR elderly*) AND NOT TS = (animal OR children).

Search strategies employed several techniques to ensure comprehensive yet focused retrieval of relevant studies. Truncation symbols (*) were used to encompass all variations of a word stem, maximizing search sensitivity. Field tags—[Title/Abstract] (PubMed), TITLE-ABS-KEY (Scopus), and TS (Topic) (Web of Science)—were applied to limit the search to the title and abstract fields, enhancing search efficiency. Additionally, studies focusing on animals or children were excluded, refining the results to research conducted on adult populations.

### 2.4 Inclusion and exclusion criteria

#### 2.4.1 Inclusion criteria

To ensure the rigor and applicability of this systematic review and meta-analysis, we included studies that met the following criteria: adult participants aged 18 years and above; investigation of the effects of creatine monohydrate supplementation, regardless of whether it was the primary or auxiliary intervention, with a systematic evaluation of its effects; assessment of changes in cognitive function (a detailed description of the specific cognitive domains assessed will be provided in a subsequent section); use of a randomized controlled trial (RCT) design, including studies employing a crossover design when appropriate; and publication between January 1, 1993, and June 5, 2024, to avoid data obsolescence and ensure the relevance of the findings.

#### 2.4.2 Exclusion criteria

In this systematic review and meta-analysis, we excluded studies if they met any of the following criteria: participants were younger than 18 years old; the study was not written in English; the study design was an *in vitro* experiment, animal experiment, or case study; the study employed a non-randomized controlled trial design such as retrospective studies, quasi-experimental designs, and existing meta-analyses; or the study presented incomplete outcome indicator information where the required data could not be supplemented by contacting the authors. These criteria ensured the quality of the studies and the completeness of the data, thereby enhancing the reliability and effectiveness of the meta-analysis.

### 2.5 Data extraction and processing

In this meta-analysis, a rigorous double-blind literature screening process was adopted, executed by two independent researchers(CX, SB). In the initial screening stage, the researchers conducted a preliminary screening based on the titles and abstracts of the literature, selecting all articles that potentially met the inclusion criteria or had unclear eligibility for full-text review. Subsequently, these two researchers independently reviewed the full texts to confirm whether the articles met the inclusion criteria for the systematic review. Any discrepancies that arose during the screening process were resolved through discussion to reach a consensus.

During the data extraction process, the researchers (CX, SB) used a standardized data extraction form specifically designed for this review to independently extract data from eligible studies, a practice consistent with established guidelines for systematic reviews and meta-analyses (16, 17). The extracted data included study characteristics (e.g., literature source, first author, and publication year), participant characteristics (e.g., age, sex, and sample size), intervention details (including dosage, duration of use, type), and outcome measures. Particular attention was given to extracting means and standard deviations for memory, attention, executive function, or processing speed from studies that assessed cognitive function using validated neuropsychological tests. For studies with incomplete data reporting or data presented only in graphical form, complete raw data were requested from the authors via email, aligning with recommendations for addressing missing data in meta-analyses (18). This approach ensured the completeness of the data and the accuracy of the analysis, thereby enhancing the reliability of the study results.

### 2.6 Quality assessment

In this study, two researchers (CX, SB) independently conducted a quality assessment of the included literature using the Cochrane Collaboration’s risk of bias assessment tool [RoB 2.0; (19)]. This tool covers several key domains, including random sequence generation, allocation concealment, blinding (including blinding of participants, researchers, and outcome assessors), data completeness, selective reporting of outcomes, and other potential biases. Each domain was rated as “low risk,” “high risk,” or “unclear” based on its risk of bias. For a study to be considered as having a low risk of bias, all domains needed to meet the criteria for low risk. Any discrepancies in judgments about the risk of bias during the assessment process were resolved through discussion. If no consensus was reached after discussion, a third researcher was involved to form a final consensus judgment.

### 2.7 Data analysis and synthesis

Data analysis was performed using Review Manager (RevMan) (Computer program, Version 5.4) and Stata (version 16; StataCorp LLC) software. The meta-analysis calculated the effects of creatine supplementation on cognitive function outcomes, including memory, attention, executive function, and processing speed. The main outcome measures were the mean scores and their standard deviations after intervention in each study. The post-intervention scores and the number of participants for each study were entered into Review Manager 5.4 software to calculate the standardized mean difference (SMD) and Hedge’s g.

The SMD was calculated as the difference in mean outcome between the creatine and placebo/control groups divided by the pooled standard deviation (20). The mathematical expression for SMD is:


$$SMD=\frac{\mathrm{Difference}\;\mathrm{in}\;\mathrm{mean}\;\mathrm{outcome}\;\mathrm{between}\;\mathrm{groups}}{\mathrm{Standard}\;\mathrm{deviation}\;\mathrm{of}\;\mathrm{outcome}\;\mathrm{among}\;\mathrm{participants}}$$

In addition to SMD, Hedge’s *g* was also calculated as a measure of effect size. Hedge’s *g* is a bias-corrected version of SMD that provides a more accurate estimate of the population effect size, especially for studies with small sample sizes (21).

When the *p* value was less than 0.1 and the heterogeneity index *I*^2^value was less than 50%, a fixed-effect model was used (22); if the *p* value was greater than or equal to 0.1 or the *I*^2^ value was greater than or equal to 50%, a random-effects model was used (23), and subgroup analysis was performed to explore potential sources of heterogeneity. The effect size for continuous data was expressed as SMD with 95% confidence intervals (CIs). The significance level was set at *p* < 0.05. For cases where the number of included studies exceeded seven, funnel plot analysis for publication bias was performed using Stata 16 software, and sensitivity analysis was conducted to assess the stability of the study results.

### 2.8 Certainty of evidence assessment

To assess the certainty of evidence, we employed the Grading of Recommendations, Assessment, Development and Evaluations (GRADE) approach. This involved evaluating the quality of evidence based on several criteria, including risk of bias, consistency of results, directness of evidence, precision of estimates, and potential publication bias. The overall certainty of the evidence for each outcome was rated as high, moderate, low, or very low.

## 3 Results

### 3.1 Literature search results

A systematic search was conducted on three major electronic databases: Web of Science (WOS), PubMed, and Scopus. A total of 2,326 research records were identified, distributed as follows: 531 from WOS, 217 from PubMed, 1,576 from Scopus, and two from other sources. After removing 471 duplicate records, 1,855 documents proceeded to the initial screening phase.

During this phase, titles and abstracts were reviewed, resulting in the exclusion of 1,837 records that were not relevant to the research topic. Consequently, 28 records were deemed eligible for full-text review.

**Figure 1:**
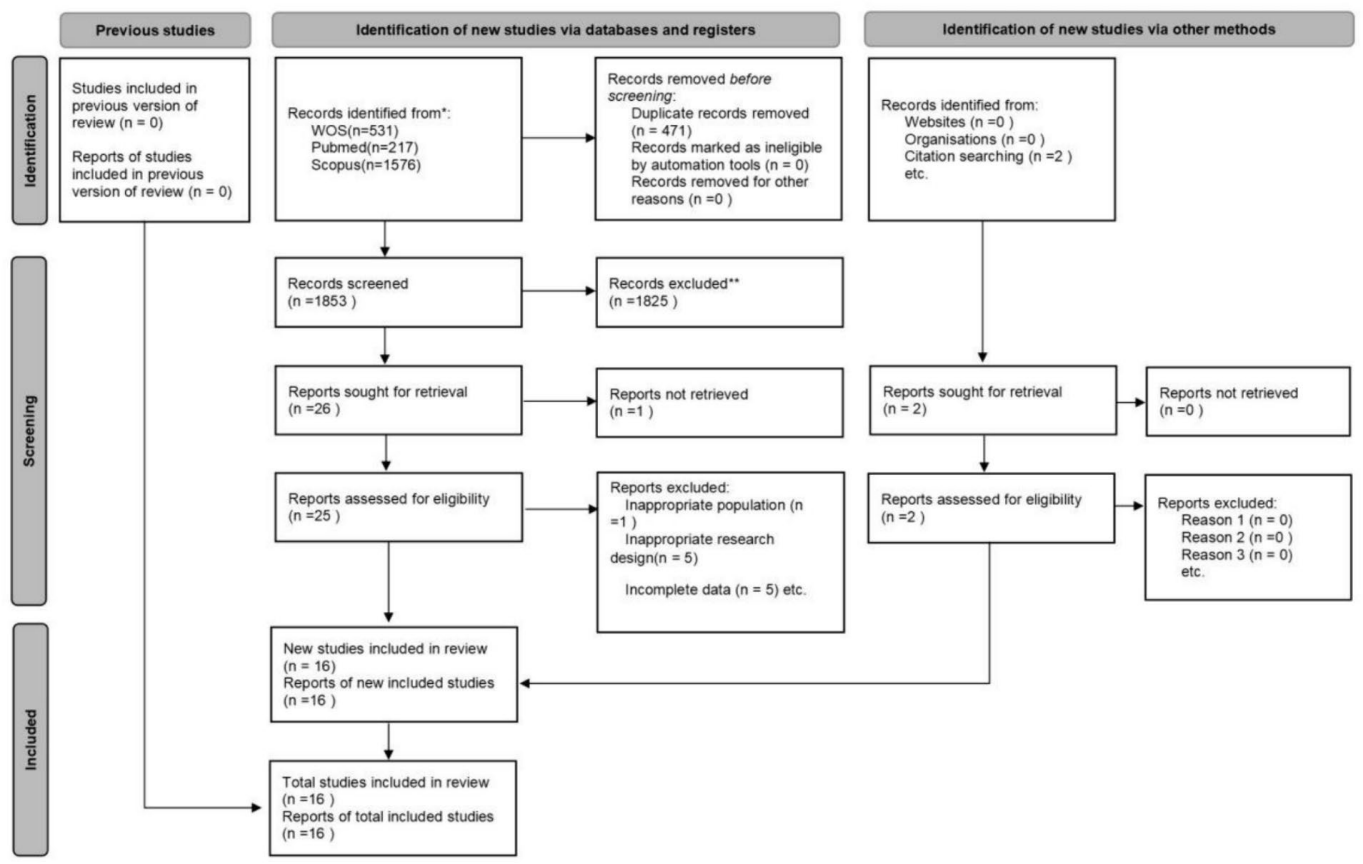
PRISMA diagram of searching and screening process.

During the full-text review process, 12 studies were excluded for not meeting the predefined inclusion criteria. The specific reasons for exclusion were: non-qualifying study population (one study), inability to retrieve full text (one study), non-compliant study design (five studies), and incomplete post-intervention data (five studies). After rigorous screening, 16 studies met the established inclusion criteria and were included in the final analysis. The detailed process of literature search and screening is illustrated in Figure 1.

### 3.2 Basic information and methodological quality assessment of included studies

#### 3.2.1 Study design

This systematic review included 16 studies with various research designs: 11 randomized controlled trials (RCTs), three double-blind crossover RCTs, and one pilot RCT. These studies were conducted in multiple countries worldwide, including four in Brazil, three in the United Kingdom, two in the United States, two in Germany, and one each in China, Iran, New Zealand, Belgium, and Israel (24–33). Table 2 presents the detailed designs and control conditions for each study.

**Table 2:** Intervention characteristics.

Author (Year)	*N* (cre/con)	Participant age: Mean (SD)	(Male/Female)	Dosage	Duration	Creatine type	Outcomes				
							Global cognitive function	Memory	Executive function	Attention	Processing speed
Alves, Merege Filho et al.	15/13	48.8 (9.1)	0/28	5 g/day	16 weeks	Creatine monohydrate	MMSE	Delay recall (naming; incidental memory; immediate; learning; and delay recall)	Trail making test (part A; part B)	Stroop test (color condition; non-color condition; color word condition)	
								Digit span test (forward order; backward order)			
Alves, Santiago, et al.	13/12	67.1 (5.1)	0/25	5 g/day	24 weeks	Creatine monohydrate	MMSE	Delay recall (naming; incidental memory; immediate; learning; delay recall)	Trail making test (part A)	Stroop test (color condition; non-color condition; color word condition)	
								Digit span test (forward order; backward order)			
Borchio et al.	10/10	29.5 (9.3)	20/0	20 g/day	7 days	Creatine monohydrate		CBT; DTT		EFT	GNGVRT; VRT
Gordji-Nejad et al.	15/15	23 (2)	8/7	0.3 g/kg/day	7.5 h	Creatine monohydrate		Word memory test; Forward memory digit span test			
Levental et al.	7/7	45.5 (8)	6/1	>3 g/day	6 months	Creatine monohydrate	MSIB Global cognitive score	MSIB memory score	MSIB executive function score		MSIB visual spatial score
Li et al.	38/37	62.65 (7.69)	50/25	10 g/day	18 months	Creatine monohydrate	MOCA				
McMorris, Jonathan P Swain, et al.	10/9	21.11 (1.85)	16/3	20 g/day	7 days	Creatine monohydrate		Forward verbal recall; Backward verbal recall; Forward spatial recall; Backward spatial recall backward			
McMorris, R.C. Harris, et al.	10/9	21.11 (1.85)	19/0	20 g/day	7 days	Creatine monohydrate		Forward number recall			Choice reaction time
McMorriset, Gregorsz Mielcarz, et al.	15/17	76.4 (8.48)	16/16	20 g/day	7 days	Creatine monohydrate		Forward number recall; Backward number recall; Forward spatial recall; Backward spatial recall; and Long-Term memory			
Moriarty et al.	20/10	21 (2.6)	11/19	10 g/day or 20 g/day	6 weeks	Creatine monohydrate		Picture sequence memory	Dimensional change card sort test		Pattern comparison test
Pires et al.	13/13	25.9 (4.6)	0/26	3 g/day	4 weeks	Creatine monohydrate		CBT; RCBT; DTT; and VFDS		EFT (% correct answers same direction; % correct answers opposite directions) EFT (Arrows in same direction; Arrows in opposite direction)	VRT; GNGVRT; ART; GNGART
Rawson et al.	11/11	20.8 (2.2)	13/9	0.3 g/kg/day	6 weeks	Creatine monohydrate					Simple reaction time
Samadi et al.	10/10	21.5 (1.5)	20/0	0.3 g/kg/day	4 weeks	Creatine monohydrate	Mathematical processing				
Sandkühler et al.	63/62	30.6 (10.1)		5 g/day	6 weeks	Creatine monohydrate	RAPM	BDS			
Turner et al.	15/15	31 (8.5)	10/5	20 g/day	7 days	Creatine monohydrate		Composite memory	Executive function; Cognitive flexibility	Complex attention	Psychomotor speed; Reaction time
Roelands, B. et al.	14/14	24 (3)	10/4	20 g/day	7 days	Creatine monohydrate				Flanker task	

#### 3.2.2 Participant characteristics

This meta-analysis included a total of 492 participants, with ages ranging from 20.8 to 76.4 years. Among the studies, three specifically targeted older adults (aged ≥60 years), while the remaining 13 focused on adults (aged 18–59 years). Regarding sex distribution, three studies included only female participants, three included only male participants, and the remaining 10 included both male and female participants. In terms of health status, 13 studies focused on healthy individuals, while three studies targeted patients with specific conditions, such as fibromyalgia, mild cognitive impairment associated with Parkinson’s disease, and chronic schizophrenia under treatment.

#### 3.2.3 Intervention details

All included studies utilized creatine supplementation, specifically creatine monohydrate, as the primary or adjunctive intervention. The duration of interventions varied across the studies: nine studies implemented interventions lasting less than 4 weeks, whereas seven studies extended the intervention period beyond 4 weeks.

#### 3.2.4 Outcome measurement indicators

All included studies provided objective measures of cognitive performance. The assessment covered various indicators including overall cognitive function, memory, executive function, attention, and processing speed.

### 3.3 Risk of bias assessment

**Figure 2:**
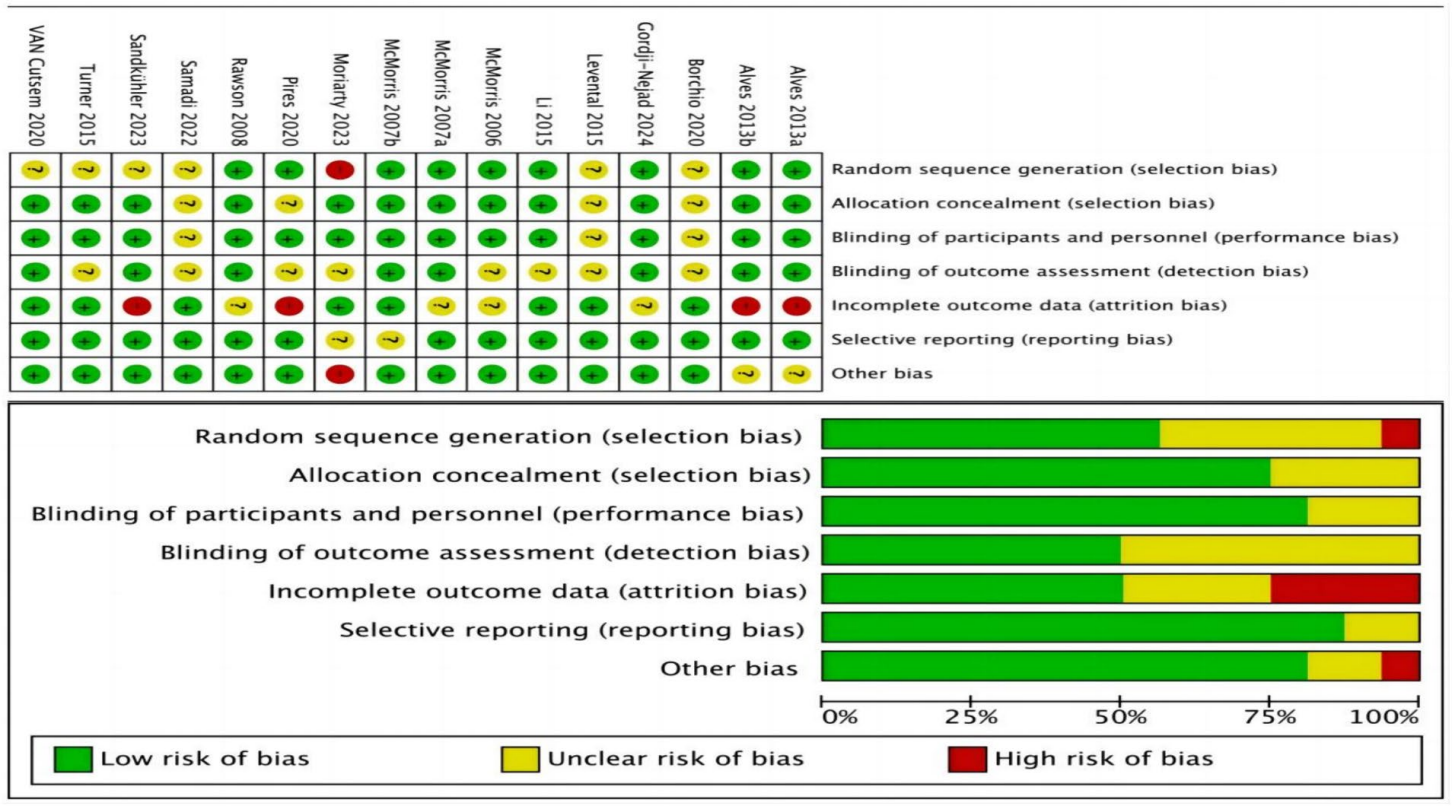
Risk of bias assessment for included studies.

The risk of bias for each included study was assessed using the Cochrane Collaboration’s tool for assessing risk of bias (RoB 2.0), implemented within the Review Manager software (RevMan 5.4). Assessments were conducted across the following bias domains: selection bias, performance bias, detection bias, attrition bias, reporting bias, and other potential sources of bias. Each domain was evaluated and classified as “low risk,” “high risk,” or “unclear risk.” A graphical representation of the risk of bias assessment results is provided in Figure 2. To evaluate publication bias, a funnel plot analysis was conducted and is presented in the Appendix. The symmetry/asymmetry of the funnel plot suggests the presence/absence of publication bias among the included studies.

### 3.4 Meta-analysis results

#### 3.4.1 Overall cognitive function

**Figure 3:**
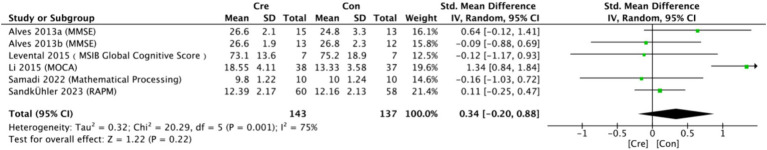
Meta-analysis forest plot of the effect of creatine supplementation on overall cognitive function.

The meta-analysis results (Figure 3) indicate that creatine supplementation does not have a significant impact on overall cognitive function. Six studies, encompassing a total of 280 participants, assessed the potential effects of creatine supplementation on overall cognitive function. The combined analysis shows an overall SMD of 0.34 (95% CI: −0.20 to 0.88), with a heterogeneity (*I*^2^) of 75% and a *Z*-value for the overall effect test of 1.22 (*p* = 0.22). Additionally, Hedges’s *g* is 0.3340 (95% CI: 0.0980–0.5700). This indicates that, although individual studies show varying degrees of effect, creatine supplementation does not have a significant positive impact on overall cognitive function when considered as a whole.

#### 3.4.2 Executive function

**Figure 4:**
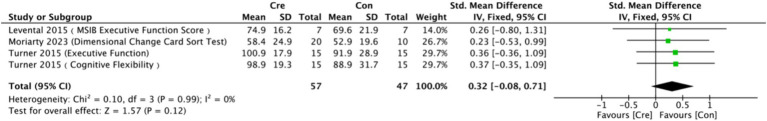
Meta-analysis forest plot of the effect of creatine supplementation on executive function scores.

The meta-analysis results (Figure 4) indicate that creatine supplementation does not have a significant impact on executive function. Four studies, encompassing a total of 104 participants, assessed the potential effects of creatine supplementation on executive function. The combined analysis shows an overall SMD of 0.32 (95% CI: −0.08 to 0.71), with a heterogeneity (*I*^2^) of 0% and a *Z*-value for the overall effect test of 1.57 (*p* = 0.12). Additionally, Hedges’s *g* is 0.3098 (95% CI: −0.0787 to 0.6983). This indicates that, although individual studies show varying degrees of effect, creatine supplementation does not have a significant positive impact on executive function when considered as a whole.

**Figure 5:**
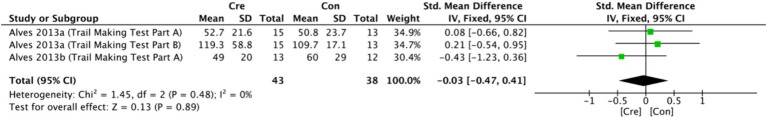
Meta-analysis forest plot of the effect of creatine supplementation on executive function time.

The meta-analysis results (Figure 5) indicate that creatine supplementation does not have a significant impact on executive function time. Three studies, encompassing a total of 81 participants, assessed the potential effects of creatine supplementation on executive function time. The combined analysis shows an overall SMD of −0.03 (95% CI: −0.47 to 0.41), with a heterogeneity (*I*^2^) of 0% and a *Z*-value for the overall effect test of 0.13 (*p* = 0.89). Additionally, Hedges’s *g* is −0.0291(95% CI: −0.4658 to 0.4076). This indicates that, although individual studies show varying degrees of effect, creatine supplementation does not have a significant positive impact on executive function time when considered as a whole.

#### 3.4.3 Attention

**Figure 6:**
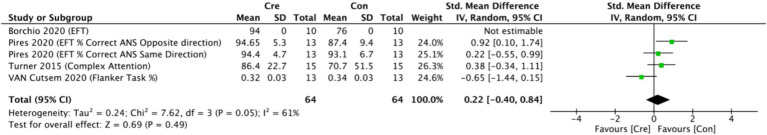
Meta-analysis forest plot of the effect of creatine supplementation on attention scores.

The meta-analysis results (Figure 6) indicate that creatine supplementation does not have a significant impact on attention scores. Four studies, encompassing a total of 128 participants, assessed the potential effects of creatine supplementation on attention scores. The combined analysis shows an overall SMD of 0.22 (95% CI: −0.40 to 0.84), with a heterogeneity (*I*^2^) of 61% and a *Z*-value for the overall effect test of 0.69 (*p* = 0.49). Additionally, Hedges’ *g* is 0.2129 (95% CI: −0.1346 to 0.5604). This indicates that, although individual studies show varying degrees of effect, creatine supplementation does not have a significant positive impact on attention scores when considered as a whole.

**Figure 7:**
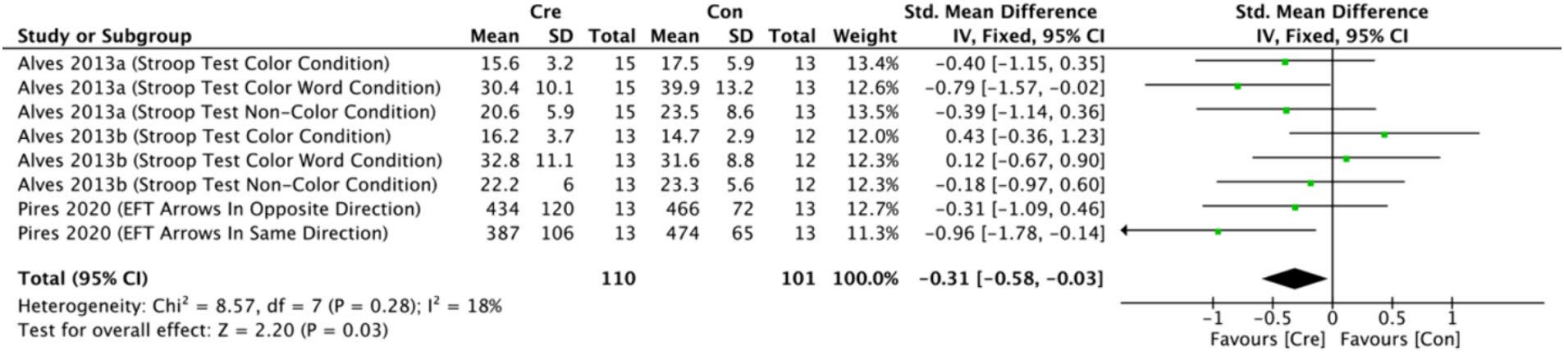
Meta-analysis forest plot of the effect of creatine supplementation on attention time.

The meta-analysis results (Figure 7) indicate that creatine supplementation has a significant positive impact on attention time. Eight studies, encompassing a total of 211 participants, assessed the potential effects of creatine supplementation on attention time. The combined analysis shows an overall SMD of −0.31 (95% CI: −0.58 to −0.03), with a heterogeneity (*I*^2^) of 18% and a *Z*-value for the overall effect test of 2.20 (*p* = 0.03). Additionally, Hedges’s *g* is −0.3004 (95% CI: −0.5719 to −0.0289). This indicates that creatine supplementation has a significant positive impact on attention time, effectively reducing the time required to complete attention tasks.

#### 3.4.4 Memory function

**Figure 8:**
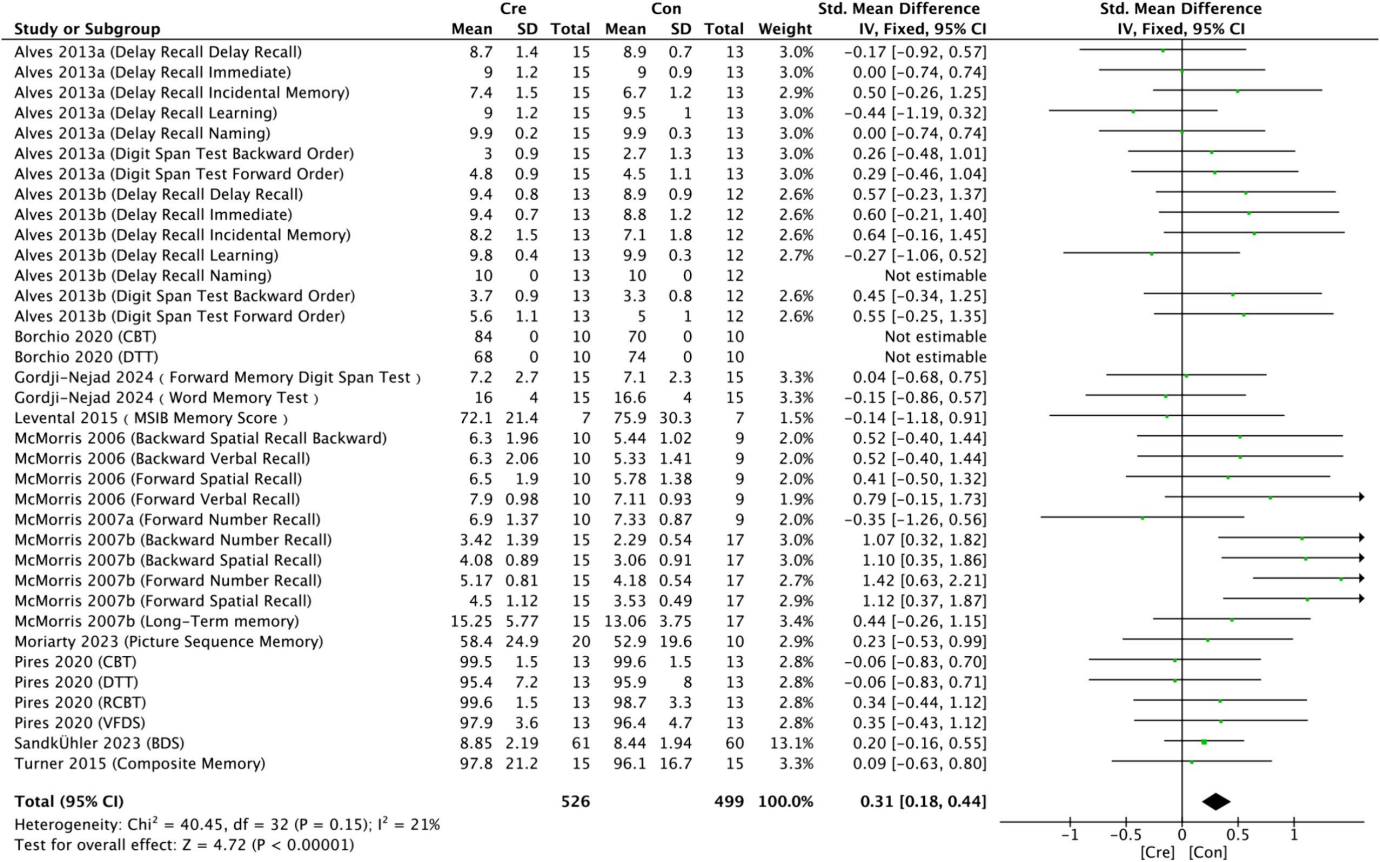
Meta-analysis forest plot of the effect of creatine supplementation on memory function.

The meta-analysis results (Figure 8) indicate that creatine supplementation has a significant positive impact on memory function. Twenty-four studies, encompassing a total of 1,000 participants, assessed the potential effects of creatine supplementation on memory function. The combined analysis shows an overall SMD of 0.31 (95% CI: 0.18–0.44), with a heterogeneity (*I*^2^) of 21% and a *Z*-value for the overall effect test of 4.72 (*p* < 0.00001). Additionally, Hedges’s *g* is 0.3003 (95% CI: 0.1778–0.4228). These results indicate that creatine supplementation has a significant positive impact on memory function, effectively improving memory performance.

#### 3.4.5 Processing speed

**Figure 9:**
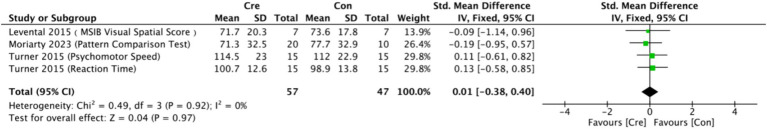
Meta-analysis forest plot of the effect of creatine supplementation on processing speed scores.

The meta-analysis results (Figure 9) indicate that creatine supplementation does not have a significant impact on processing speed scores. Four studies, encompassing a total of 104 participants, assessed the potential effects of creatine supplementation on processing speed scores. The combined analysis shows an overall SMD of 0.01 (95% CI: −0.38 to 0.40), with a heterogeneity (*I*^2^) of 0% and a *Z*-value for the overall effect test of 0.04 (*p* = 0.97). Additionally, Hedges’ *g* is 0.0097 (95% CI: −0.3764 to 0.3958). This indicates that, although individual studies show varying degrees of effect, creatine supplementation does not have a significant positive impact on processing speed scores when considered as a whole.

**Figure 10:**
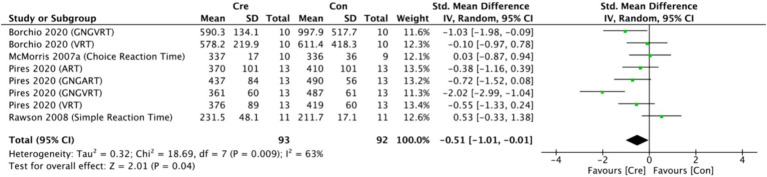
Meta-analysis forest plot of the effect of creatine supplementation on processing speed time.

The meta-analysis results (Figure 10) indicate that creatine supplementation has a significant positive impact on processing speed time. Eight studies, encompassing a total of 185 participants, assessed the potential effects of creatine supplementation on processing speed time. The combined analysis shows an overall SMD of −0.51 (95% CI: −1.01 to −0.01), with a heterogeneity (*I*^2^) of 63% and a *Z*-value for the overall effect test of 2.01 (*p* = 0.04). Additionally, Hedges’s *g* is −0.4916 (95% CI: −0.7852 to −0.1980). These results indicate that creatine supplementation has a significant positive impact on processing speed time, effectively reducing the time required to complete processing speed tasks.

### 3.5 Subgroup analysis results

#### 3.5.1 Subgroup analysis of attention

The subgroup analysis results indicate varying effects of creatine supplementation on attention time across different subgroups, specifically evaluating health status, age, and intervention duration (Table 3).

**Table 3:** Subgroup analysis results for attention time.

Attention (time)								
Covariate	Group	SMD	Lower 95% CI	Upper 95% CI	p	Heterogeneity (*I*^2^)	*p* between groups	Heterogeneity between groups (*I*^2^)
Population	8	−0.31	−0.61	0			0.28	13.80%
Health	5	−0.18	−0.62	0.27	0.44	38%		
Illness	3	−0.52	−0.96	−0.08	0.02	0%		
Age (years)	8	−0.31	−0.61	0			0.02	81%
>60	3	0.12	−0.34	0.58	0.61	0%		
18–60	5	−0.55	−0.9	−0.21	0.002	0%		
Intervention duration	8	−0.31	−0.61	0			0.26	21%
Less than 4 weeks	2	−0.62	−1.25	0.01	0.61	21%		
More than 4 weeks	6	−0.21	−0.61	0.13	0.23	14%		

Firstly, when grouped by health status, the overall SMD from eight studies was −0.31 (95% CI: −0.61 to 0), with heterogeneity (*I*^2^) of 13.80%. In healthy individuals, five studies showed an SMD of −0.18 (95% CI: −0.62 to 0.27), with *I*^2^ of 38% and *p* value of 0.44, indicating no significant effect. However, in individuals with illnesses, three studies reported a significant effect with an SMD of −0.52 (95% CI, −0.96 to −0.08), *I*^2^ of 0%, and *p* value of 0.02.

Secondly, by age groups, the overall SMD from eight studies was −0.31 (95% CI: −0.61 to 0), with *I*^2^ of 81% and *p* value of 0.02. In participants over 60 years old, three studies showed an SMD of 0.12 (95% CI: −0.34 to 0.58), with *I*^2^ of 0% and *p* value of 0.61, indicating no significant effect. Conversely, in the 18–60 years age group, five studies found a significant positive effect with an SMD of −0.55 (95% CI: −0.90 to −0.21), with *I*^2^ of 0% and *p* value of 0.002.

Lastly, when analyzed by intervention duration, the overall SMD from eight studies was −0.31 (95% CI: −0.61 to 0), with *I*^2^ of 21% and *p* value of 0.26. For interventions lasting less than 4 weeks, two studies reported an SMD of −0.62 (95% CI: −1.25 to 0.01), with *I*^2^ of 21% and *p* value of 0.61, showing no significant effect. For interventions longer than 4 weeks, six studies showed an SMD of −0.21 (95% CI: −0.61 to 0.13), with *I*^2^ of 14% and *p* value of 0.23, also indicating no significant effect.

Overall, these findings suggest that creatine supplementation significantly reduces attention time in individuals with illnesses and in the 18–60 years age group, but not in healthy individuals or those over 60 years old. The duration of the intervention does not significantly alter the effects, whether short-term or long-term.

#### 3.5.2 Subgroup analysis of processing speed time

The subgroup analysis results indicate that the effects of creatine supplementation on processing speed time may have sex-specific responses (Table 4).

**Table 4:** Study on the effects of creatine supplements on processing speed time in different gender populations.

Processing speed (time)								
Covariate	Group	SMD	Lower 95% CI	Upper 95% CI	p	Heterogeneity (*I*^2^)	*p* between groups	Heterogeneity between groups (*I*^2^)
Sex	7	−0.51	−1.01	−0.01			0.04	69%
Male	3	−0.35	−0.99	0.30	0.29	34%		
Female	4	−0.87	−1.53	−0.21	0.01	60%		

Firstly, the overall SMD from eight studies, grouped by sex, was −0.51 (95% CI: −1.01 to −0.01), with heterogeneity (*I*^2^) of 69% and a *p* value of 0.04. In male participants, three studies showed an SMD of −0.35 (95% CI: −0.99 to 0.30), with *I*^2^ of 34% and a *p* value of 0.29, indicating no significant effect. Conversely, in female participants, four studies reported a significant effect with an SMD of −0.87 (95% CI: −1.53 to −0.21), with *I*^2^ of 60% and a *p*-value of 0.01.

Overall, these findings suggest that creatine supplementation significantly reduces processing speed time in female participants but does not have a significant effect in male participants. This indicates that the response to creatine supplementation in improving processing speed time may have sex-specific characteristics.

### 3.6 Sensitivity analysis

**Figure 11:**
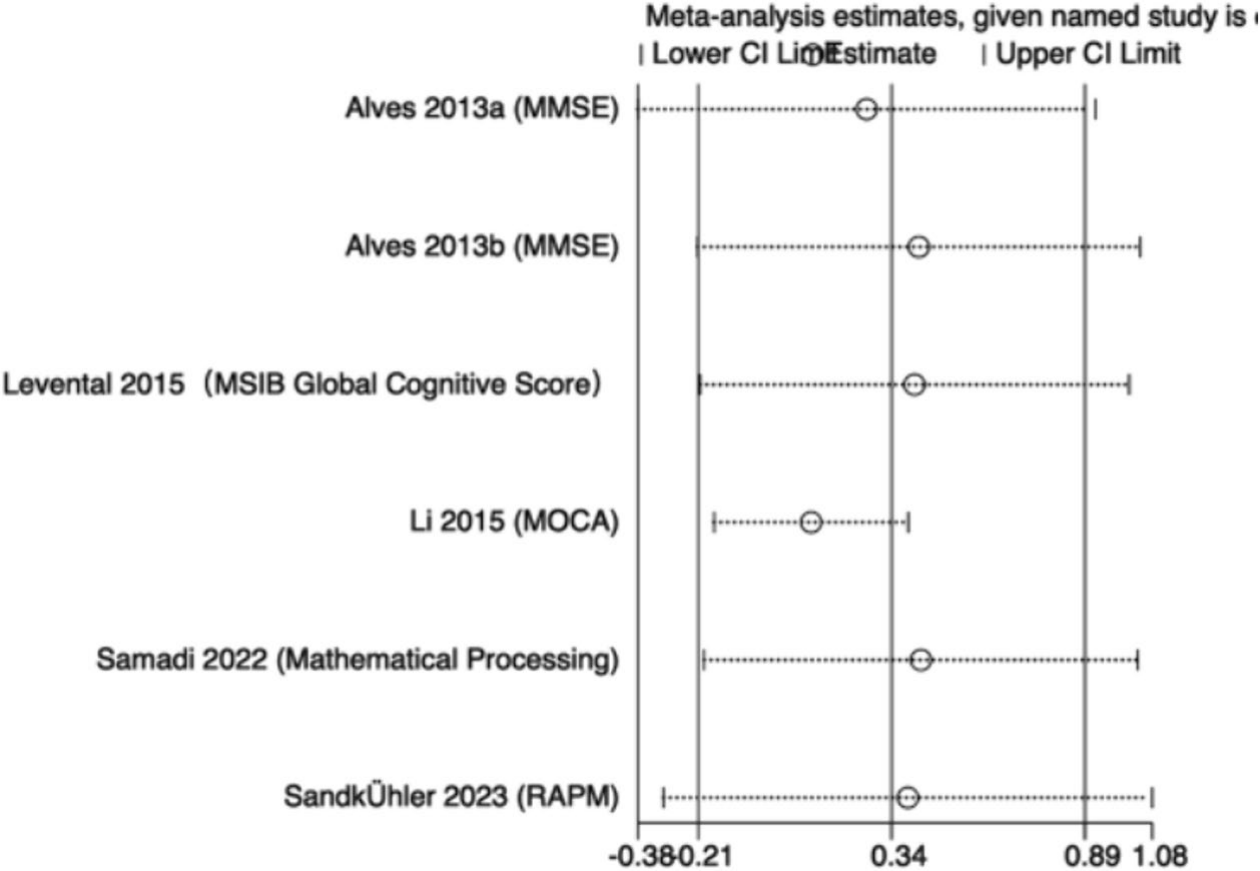
Sensitivity analysis chart of global cognitive function.

**Figure 12:**
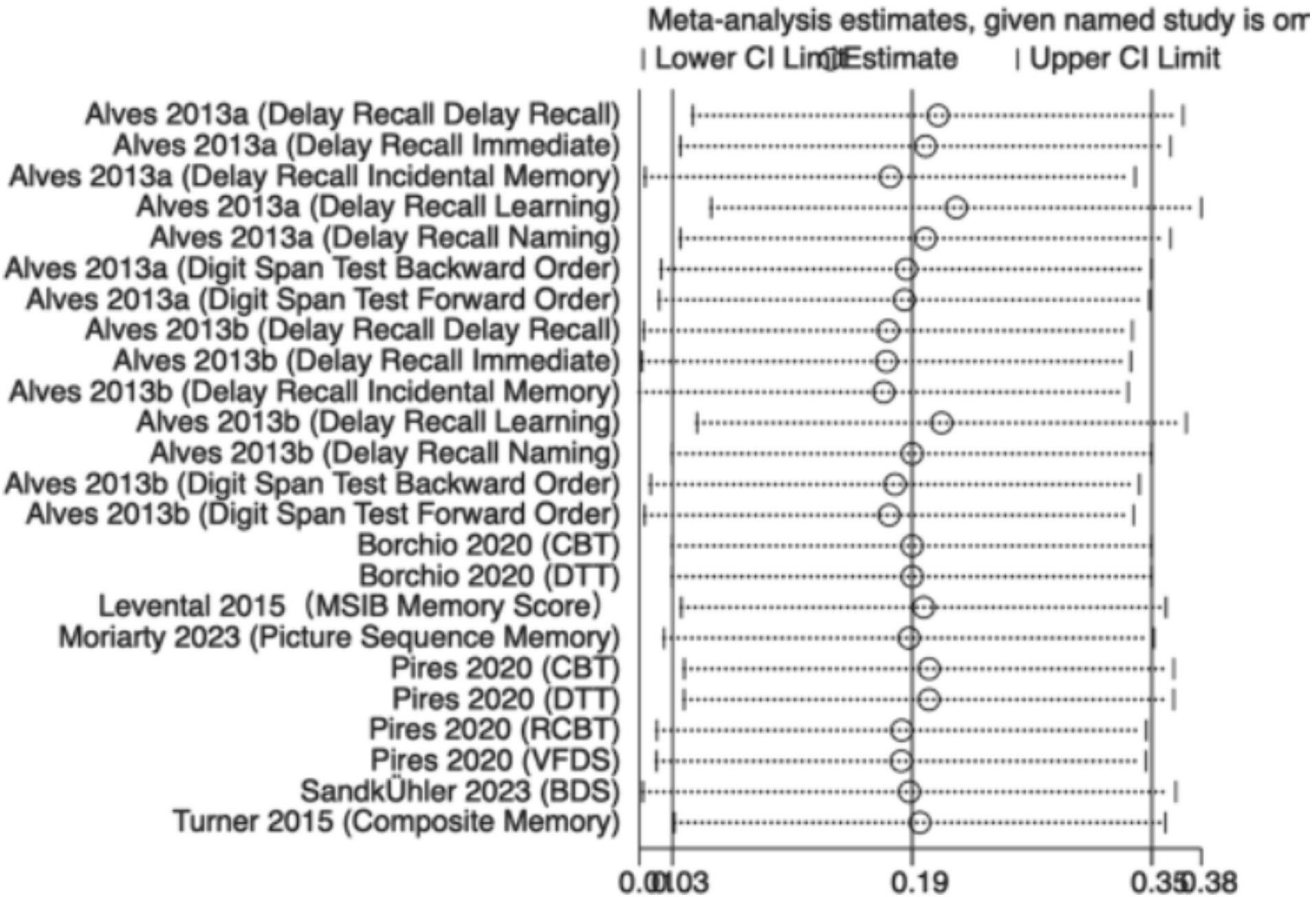
Sensitivity analysis chart of memory.

**Figure 13:**
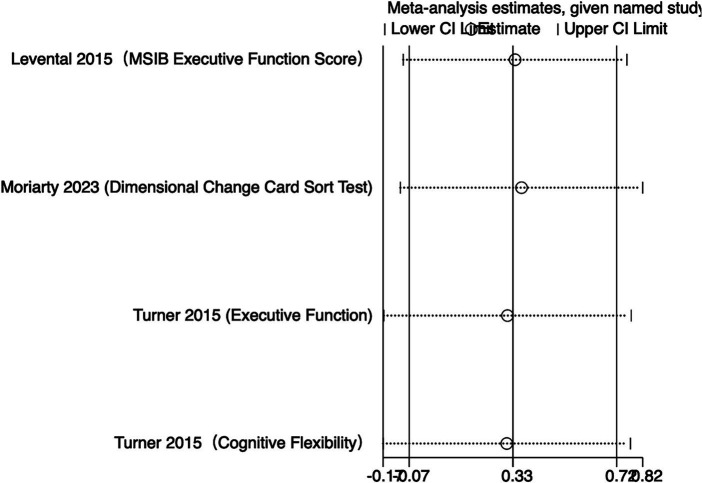
Sensitivity analysis chart of executive function.

**Figure 14:**
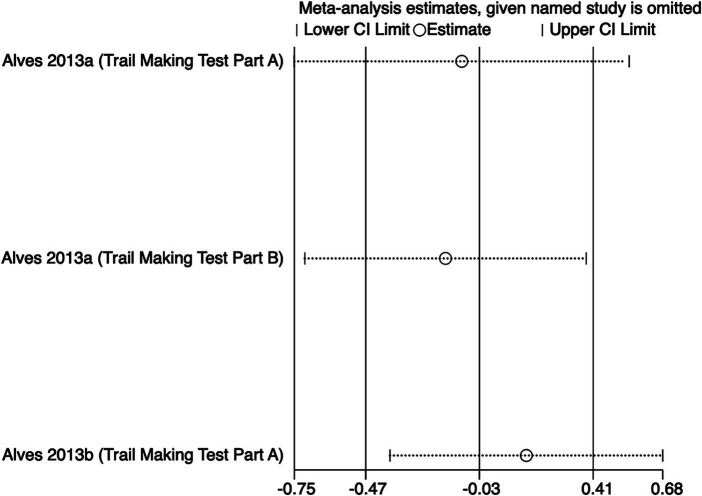
Sensitivity analysis chart of executive function time.

**Figure 15:**
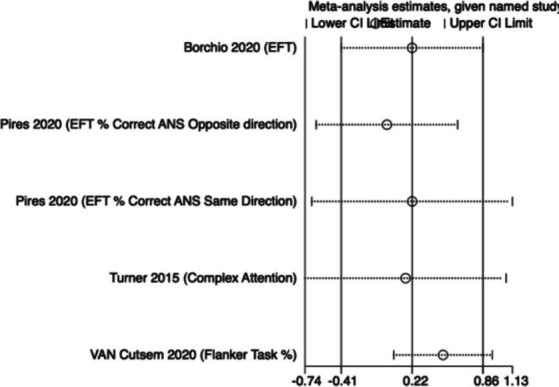
Sensitivity analysis chart of attention.

**Figure 16:**
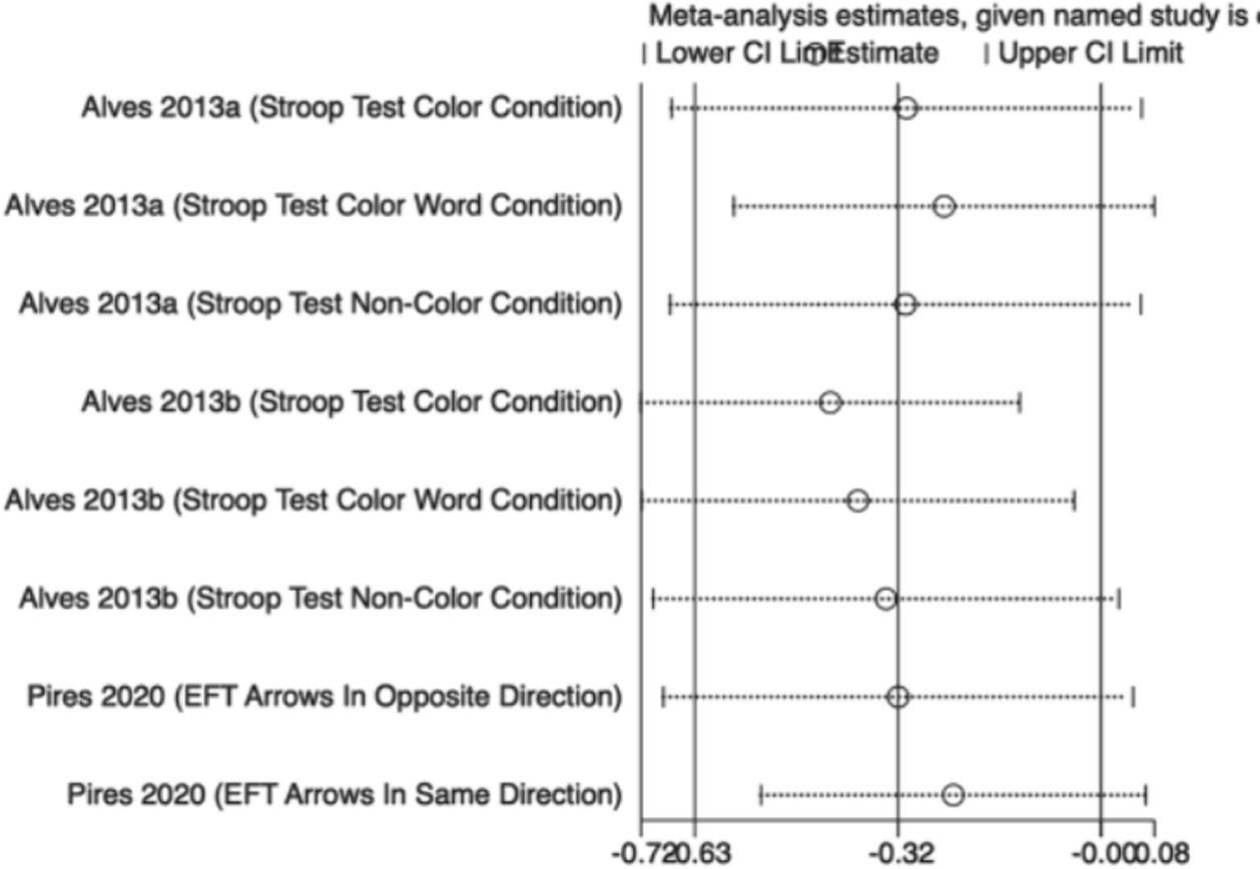
Sensitivity analysis chart of attention time.

**Figure 17:**
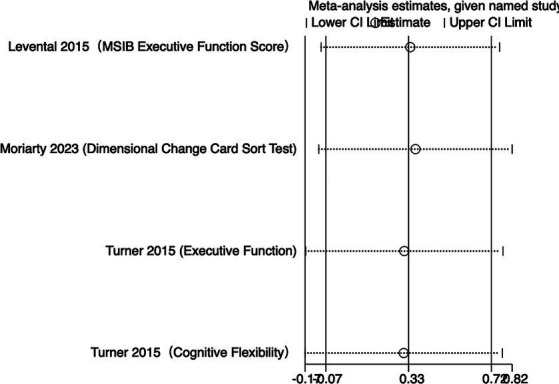
Sensitivity analysis chart of processing speed.

**Figure 18:**
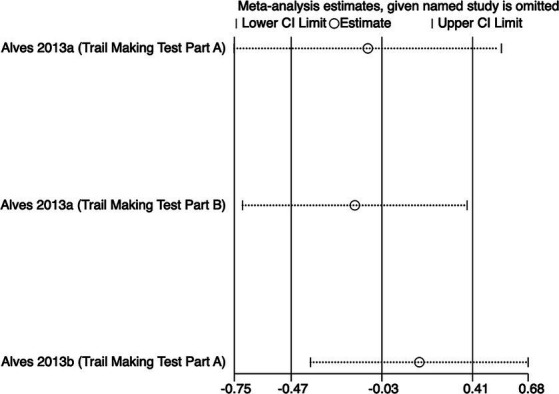
Sensitivity analysis chart of processing speed time.

Sensitivity analyses were conducted on the research findings for overall cognitive function and specific cognitive domain indicators to assess the stability of the results. These analyses were performed by sequentially excluding each study and examining whether the combined effect size estimates of the remaining studies still fell within the 95% confidence interval range of the overall combined effect size. The results revealed that removing any single study did not significantly influence the overall conclusions, further confirming the robustness of the research findings. The corresponding sensitivity analysis plots provided detailed illustrations of the analysis results for overall cognitive function and specific cognitive domain indicators (Figures 11–18).

### 3.7 Egger’s test

Egger’s test was used to quantitatively analyze publication bias, and the results are presented in Table 5. The *p* values for Global cognitive function (*p* = 0.771), Memory (*p* = 0.494), Executive function (*p* = 0.546), Executive function time (*p* = 0.147), Attention (*p* = 0.979), Attention time (*p* = 0.974), Processing speed (*p* = 0.543), and Processing speed time (*p* = 0.348) were all significantly higher than the 0.05 threshold, indicating no statistically significant publication bias.

**Table 5:** Egger’s test results for the meta-analysis.

Outcomes	Std_Eff	Coef.	Std.Err	t	p	95%CI
Global cognitive function	Slope	0.6156409	0.7533144	0.82	0.46	−1.475895 to 2.707177
	Bias	−0.7782632	2.496782	−0.31	0.771	−7.710442 to 6.153916
Memory	Slope	0.0695127	0.3543886	0.20	0.846	−0.6532676 to 0.792293
	Bias	0.652748	0.9347061	0.70	0.490	−1.253598 to 2.559094
Executive function	Slope	0.5291055	0.2851093	1.86	0.205	−0.6976209 to 1.755832
	Bias	−0.5121732	0.7099642	−0.72	0.546	−3.566903 to 2.542556
Executive function time	Slope	8.736751	2.064796	4.23	0.148	−17.49897 to 34.97248
	Bias	−22.62588	5.325404	−4.25	0.147	−90.29155 to 45.03979
Attention	Slope	−0.0368459	8.946358	0	0.997	−38.52992 to 38.45623
	Bias	0.6623473	22.68819	0.03	0.979	−96.95707 to 98.28176
Attention time	Slope	−0.0988147	6.466409	−0.02	0.988	−0.15.92155 to 15.72392
	Bias	−0.5551808	16.27624	−0.03	0.974	−40.38171 to 39.27135
Processing speed	Slope	0.4612439	0.6309956	0.73	0.541	−2.253711 to 3.176199
	Bias	−1.14873	1.580664	−0.73	0.543	−7.949778 to 5.652318
Processing speed time	Slope	2.875177	3.325391	0.86	0.420	−5.261762 to 11.01212
	Bias	−7.768328	7.624275	−1.02	0.348	−26.42426 to 10.8876

### 3.8 Certainty of evidence

The GRADE assessment indicated that the certainty of evidence for the impact of creatine supplementation on memory was moderate. For processing speed, the certainty was rated as low. The certainty for other cognitive domains, such as overall cognitive function, executive function, and attention, ranged from low to very low. Detailed GRADE assessments are provided in the Supplementary material.

## 4 Discussion

This systematic review with meta-analysis comprehensively investigated the effects of creatine monohydrate supplementation on cognitive function, focusing on key cognitive domains such as memory, executive function, attention, and information processing speed. As a widely used nutritional supplement among athletes, creatine has been well-recognized for its effectiveness in enhancing muscle strength and endurance. However, its potential benefits in improving cognitive function in both the general adult population and specific groups, such as the elderly and patients with neurodegenerative diseases, had not been thoroughly elucidated. In the brain, creatine may improve cognitive performance by increasing cellular energy reserves and reducing oxidative stress, which is particularly important in tasks requiring high cognitive processing. This study not only examined the cognitive responses of the general adult population but also specifically considered the cognitive function of the elderly and patients with neurodegenerative diseases, aiming to provide more scientific evidence and clinical guidance for the application of creatine in these populations.

The findings demonstrated that creatine supplementation significantly improved memory (SMD = 0.31; 95% CI: 0.17–0.44; *I*^2^ = 23%; *p* < 0.00001). This result aligns with the study by Rawson and Volek, who reported that creatine enhances memory performance in complex tasks (34). Similarly, Avgerinos et al.’s systematic review noted positive effects of creatine on cognitive and memory functions, especially in tasks requiring high cognitive control (15).

The mechanisms underlying creatine’s enhancement of memory likely involve several biological pathways. Firstly, creatine increases the energy supply to brain cells, particularly in the form of phosphocreatine (PCr), which is crucial for maintaining cellular ATP levels in the energy-demanding brain (35). Secondly, creatine may enhance memory by improving neurotransmitter function, such as by increasing the synthesis of neurotransmitters like acetylcholine (36). Additionally, creatine may function as a neuromodulator, potentially affecting synaptic efficacy and plasticity, which are vital for learning and memory processes. Furthermore, creatine may exhibit neuroprotective properties by mitigating oxidative stress damage to brain cells (37). For instance, studies have shown that creatine is present in synaptic vesicles, released upon stimulation, and can be taken up by synaptosomes and synaptic vesicles, thereby enhancing neurotransmission (36). Moreover, research has indicated that creatine supplementation may significantly reduce processing speed time in women, suggesting potential sex-specific effects in improving cognitive function (38).

Although creatine supplementation did not achieve statistical significance in improving overall executive function, it may provide some benefits in specific types of executive function tests, particularly those requiring high cognitive demand. Benton and Donohoe noted that creatine supplementation appears to enhance cognitive processing speed and accuracy in complex cognitive tasks (39). The lack of significant effects may be attributed to the small sample size and limited number of studies analyzed in the meta-analysis. This suggests that future research should use more standardized and sensitive testing methods to better capture the potential impact of creatine on executive function.

Creatine supplementation did not significantly improve attention scores but demonstrated potential benefits in processing speed. Rae found that creatine supplementation could enhance the speed and accuracy of cognitive tasks, particularly in continuous memory tasks and other tasks requiring rapid information processing (40). This indirect effect may result from creatine’s enhancement of brain energy metabolism and increased efficiency of brain cells. However, attention involves multiple complex cognitive processes, and the lack of observed significant effects may be due to the types of tests used, sample sizes, or the sensitivity of the tests. Additionally, the small sample size and limited number of studies in the meta-analysis may contribute to the lack of significant effects. This indicates that future research should employ more specific and sensitive testing methods to better evaluate the impact of creatine on attention.

The significant improvement in processing speed time (SMD = −0.51; 95% CI: −1.01 to −0.02; I^2^ = 63%; *p* = 0.04) suggests that creatine supplementation can markedly accelerate information processing speed. This outcome may be influenced by the larger number of studies included in the meta-analysis. Future research should continue to explore the underlying mechanisms of this effect. Avgerinos et al.’s systematic review further supports the potential benefits of creatine in enhancing brain energy metabolism and cognitive function (15). This finding holds particular importance for individuals who require rapid information processing, such as students and professionals, as well as elderly individuals experiencing a decline in cognitive speed.

The effects of creatine supplementation exhibit significant variability across different populations. For individuals with medical conditions, creatine supplementation can effectively improve energy supply, showing significant potential benefits (41). This suggests that creatine may be a promising adjunctive treatment, especially for patients with neurodegenerative diseases and associated cognitive impairments. In age-stratified analysis, adults aged 18–60 showed significant effects from creatine supplementation, whereas the effects were not significant in individuals over 60 years old, possibly due to age-related physiological changes (42). This highlights the need for future research to further explore the effects of creatine across different age groups and to optimize supplementation strategies to meet the needs of various populations.

Regarding the impact of intervention duration on the effects of creatine supplementation on cognitive function, our results indicate that short-term interventions (less than 4 weeks) and long-term interventions (more than 4 weeks) did not show significant differences in improving cognitive function. This finding suggests that the effects of creatine on cognitive function may reach a saturation point within a certain period, implying that extending the intervention duration does not provide additional benefits. This result is crucial for optimizing creatine supplementation strategies, indicating that the cognitive enhancement effects of creatine may plateau within a specific time frame. For example, Roelands et al. (43) found that participants’ working memory improved after 6 weeks of creatine supplementation. Similarly, a systematic review by Avgerinos et al. (15) reported that short-term, high-dose supplementation (e.g., 20 g per day for 5 days) also showed significant effects on cognitive function tests. These studies support our conclusion that the effects of creatine on cognitive function may reach a saturation point in the short term, and extending the intervention duration does not provide additional benefits.

To evaluate the robustness of our overall conclusions, we conducted sensitivity analyses. The results indicated that even when excluding individual studies or changing the analysis methods, the overall conclusions remained consistent. This demonstrates the high robustness of our findings, which are not easily influenced by individual studies or analytical methods. Additionally, Egger’s test results indicated no significant publication bias in our study. Publication bias refers to the tendency for studies with positive results to be published more readily, while studies with negative results may be overlooked or difficult to publish, leading to systematic bias in the literature (44). Egger’s test results enhance the credibility and scientific validity of our findings, indicating that the studies included in our analysis did not show obvious selection bias during publication. However, given the inherent uncertainties in all biological phenomena, we cannot draw entirely definitive conclusions. The results should be discussed from the perspective of potential biases to provide a more nuanced understanding of methodological consistency and to enhance the robustness of our findings.

This study provides substantial evidence on the effects of creatine monohydrate supplementation on cognitive function through systematic review and meta-analysis. The results indicate that creatine supplementation significantly improves memory and information processing speed and shows positive effects in specific executive function tests. These findings support the potential of creatine as a cognitive enhancer, particularly in tasks requiring high cognitive processing. Creatine may enhance cognitive function through various mechanisms, such as increasing brain energy supply, regulating neurotransmitter levels, and improving neuronal function. These findings are valuable for individuals seeking to enhance cognitive performance and for clinicians developing intervention strategies.

However, several limitations must be acknowledged. Firstly, the included studies exhibited heterogeneity in design, sample size, and testing methods, which may affect the interpretation and generalizability of the results. The relatively small sample sizes limit the statistical power and generalizability of the conclusions. Future research should adopt more consistent and standardized methodologies and increase sample sizes to improve the comparability and reliability of the results. Secondly, most included studies focused on healthy adults, with limited evidence available for specific populations such as the elderly and patients with neurodegenerative diseases. The cognitive function of these populations may be influenced by various factors, and the effects of creatine supplementation may differ. Future research should further explore the effects of creatine in these specific populations and investigate the safety and tolerability of long-term supplementation.

Despite these limitations, the results of this study provide promising evidence for creatine as a cognitive enhancer, particularly in improving memory and information processing speed. Notably, these findings specifically support creatine monohydrate as an effective form of supplementation. This evidence offers a scientific basis for the application of creatine in cognitive enhancement and provides direction for future research. Future studies should aim to optimize creatine supplementation strategies, including exploring the optimal dosage, supplementation duration, and long-term effects, to maximize its cognitive benefits. Additionally, further research is needed to elucidate the mechanisms by which creatine affects cognitive function and to investigate its interactions with other cognitive interventions, such as cognitive training and other nutritional supplements.

## 5 Conclusion

Current evidence suggests that creatine monohydrate supplementation may confer beneficial effects on cognitive function in adults, particularly in the domains of memory, attention time, and information processing speed. Larger robust clinical trials are warranted to further validate these findings. Furthermore, future research should investigate the influence of different populations and intervention durations on the effects of creatine monohydrate supplementation, as well as elucidate the precise mechanisms underlying its potential cognitive-enhancing properties.

## Data availability statement

The original contributions presented in the study are included in the article/Supplementary material, further inquiries can be directed to the corresponding author.

## Author contributions

CX: Conceptualization, Data curation, Methodology, Resources, Visualization, Writing – original draft. SB: Formal analysis, Methodology, Writing – original draft. WZ: Formal Analysis, Methodology, Writing – original draft. LL: Conceptualization, Funding acquisition, Writing – original draft, Writing – review & editing.
