# Corrigendum: The effects of creatine supplementation on cognitive function in adults: a systematic review and meta-analysis

**DOI:** 10.3389/fnut.2025.1570800

**Published:** 2025-02-17

**Authors:** Chen Xu, Siyuan Bi, Wenxin Zhang, Lin Luo

**Affiliations:** School of Physical Education, Guizhou Normal University, Guiyang, China

**Keywords:** creatine, cognitive function, brain health, neuropsychological tests, randomized controlled trials

In the published article, there was an error due to a translation from Chinese to English. A correction has been made to **Results**, *Section 3.4.3 Attention*, paragraph one.

The paragraph previously read as: “The meta-analysis results (Figure 6) indicate that creatine supplementation does not have a significant impact on attention. Four studies, encompassing a total of 128 participants, assessed the potential effects of creatine supplementation on attention. The combined analysis shows an overall SMD of 0.22 (95% CI: −0.40 to 0.84), with a heterogeneity (*I*^2^) of 61% and a Z-value for the overall effect test of 0.69 (*p* = 0.49). Additionally, Hedges's g is 0.2129 (95% CI: −0.1346 to 0.5604). This indicates that, although individual studies show varying degrees of effect, creatine supplementation does not have a significant positive impact on attention when considered as a whole.”

The corrected paragraph appears below:

“The meta-analysis results (Figure 6) indicate that creatine supplementation does not have a significant impact on attention scores. Four studies, encompassing a total of 128 participants, assessed the potential effects of creatine supplementation on attention scores. The combined analysis shows an overall SMD of 0.22 (95% CI: −0.40 to 0.84), with a heterogeneity (*I*^2^) of 61% and a Z-value for the overall effect test of 0.69 (*p* = 0.49). Additionally, Hedges' g is 0.2129 (95% CI: −0.1346 to 0.5604). This indicates that, although individual studies show varying degrees of effect, creatine supplementation does not have a significant positive impact on attention scores when considered as a whole.”

In the published article, there was an error due to the translation from Chinese to English. A correction has been made to **Results**, *Section 3.4.5 Processing speed*, paragraph one.

The paragraph previously read as: “The meta-analysis results (Figure 9) indicate that creatine supplementation does not have a significant impact on processing speed. Four studies, encompassing a total of 104 participants, assessed the potential effects of creatine supplementation on processing speed. The combined analysis shows an overall SMD of 0.01 (95% CI: −0.38 to 0.40), with a heterogeneity (*I*^2^) of 0% and a *Z*-value for the overall effect test of 0.04 (*p* = 0.97). Additionally, Hedges's *g* is 0.0097 (95% CI: −0.3764 to 0.3958). This indicates that, although individual studies show varying degrees of effect, creatine supplementation does not have a significant positive impact on processing speed when considered as a whole.”

The corrected paragraph appears below:

“The meta-analysis results (Figure 9) indicate that creatine supplementation does not have a significant impact on processing speed scores. Four studies, encompassing a total of 104 participants, assessed the potential effects of creatine supplementation on processing speed scores. The combined analysis shows an overall SMD of 0.01 (95% CI: −0.38 to 0.40), with a heterogeneity (*I*^2^) of 0% and a *Z*-value for the overall effect test of 0.04 (*p* = 0.97). Additionally, Hedges' *g* is 0.0097 (95% CI: −0.3764 to 0.3958). This indicates that, although individual studies show varying degrees of effect, creatine supplementation does not have a significant positive impact on processing speed scores when considered as a whole.”

The authors apologize for this error and state that this does not change the scientific conclusions of the article in any way. The original article has been updated.

