# Supplementary material for: The effects of creatine supplementation on cognitive function in adults: a systematic review and meta-analysis
Source: Front Nutr. 2024 Jul 12;11:1424972. doi: 10.3389/fnut.2024.1424972 (PMC11275561; doi:10.3389/fnut.2024.1424972)
Supplement: Supplementary file 1 [file Presentation_1.pdf]

Appendix 1 Begg's funnel plot

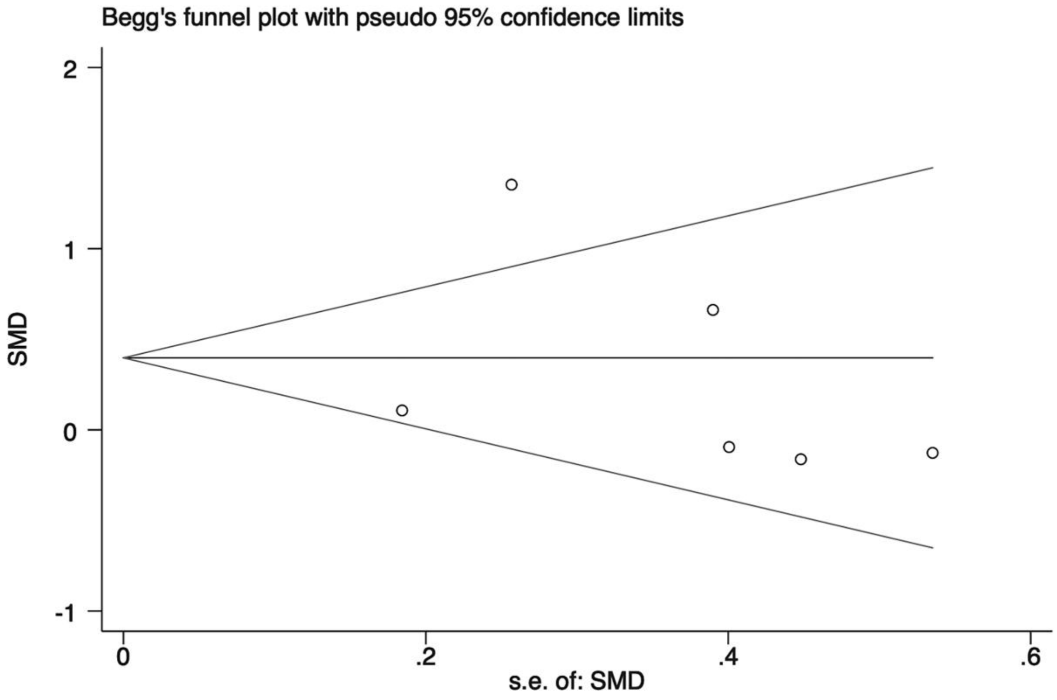

Figure1 Begg test of global cognitive function

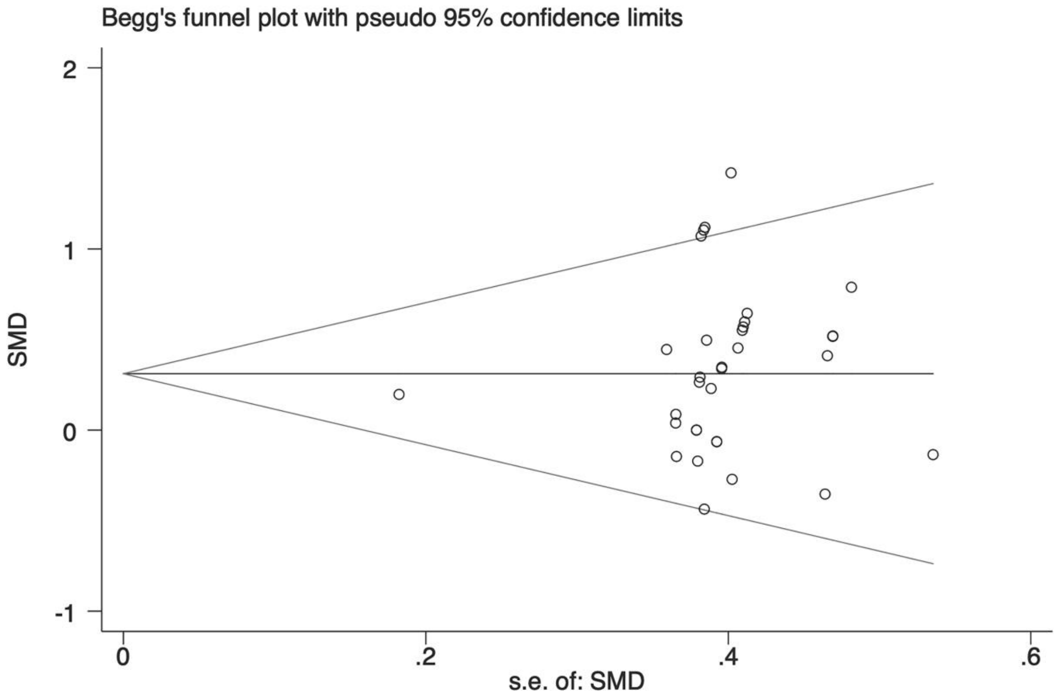

Figure2 Begg test of memory

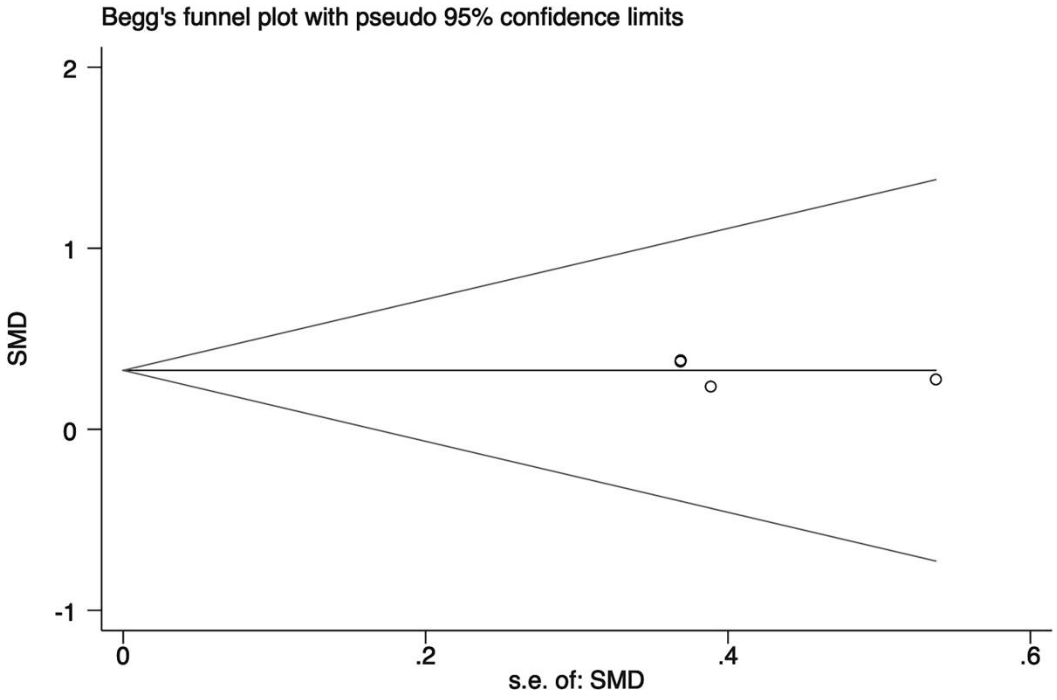

Figure3 Begg test of executive function

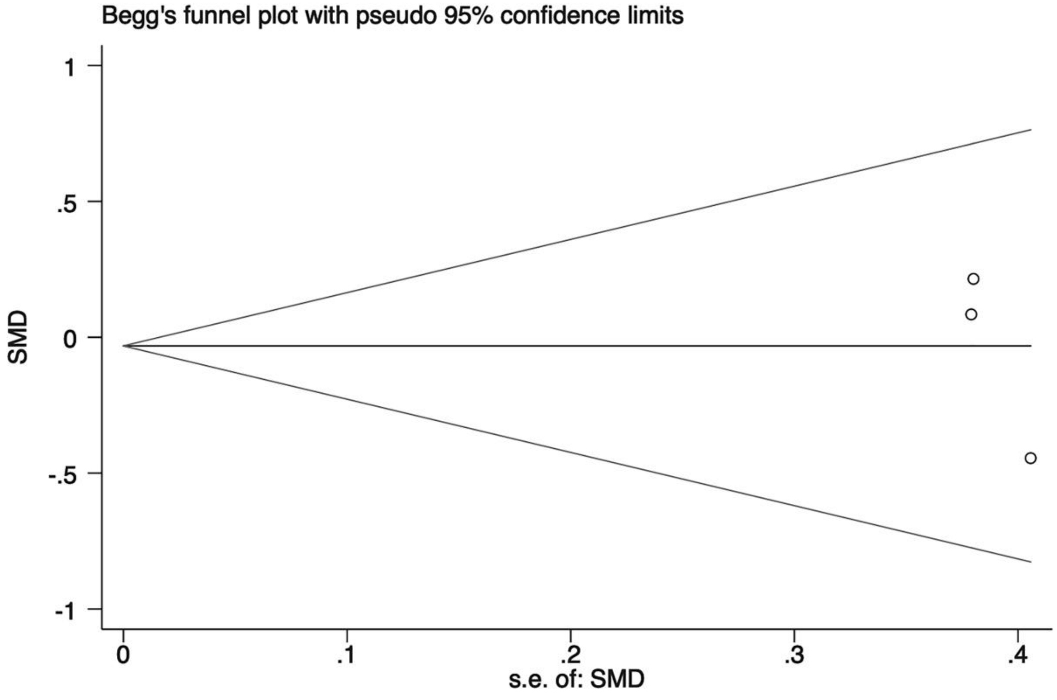

Figure4 Begg test of executive function time

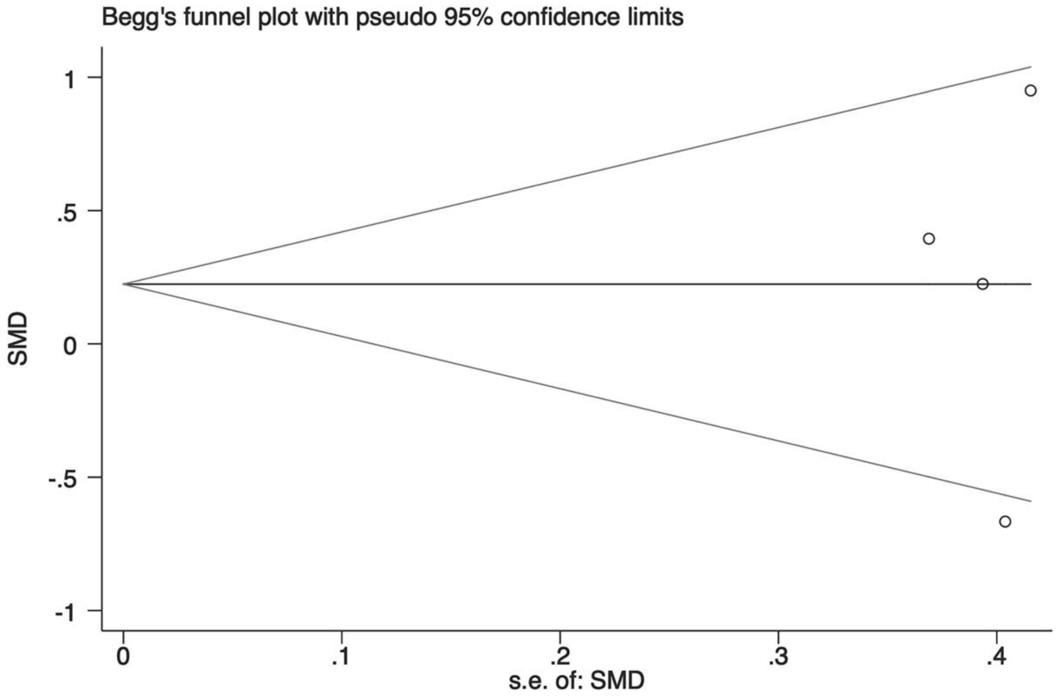

Figure5 Begg test of attention

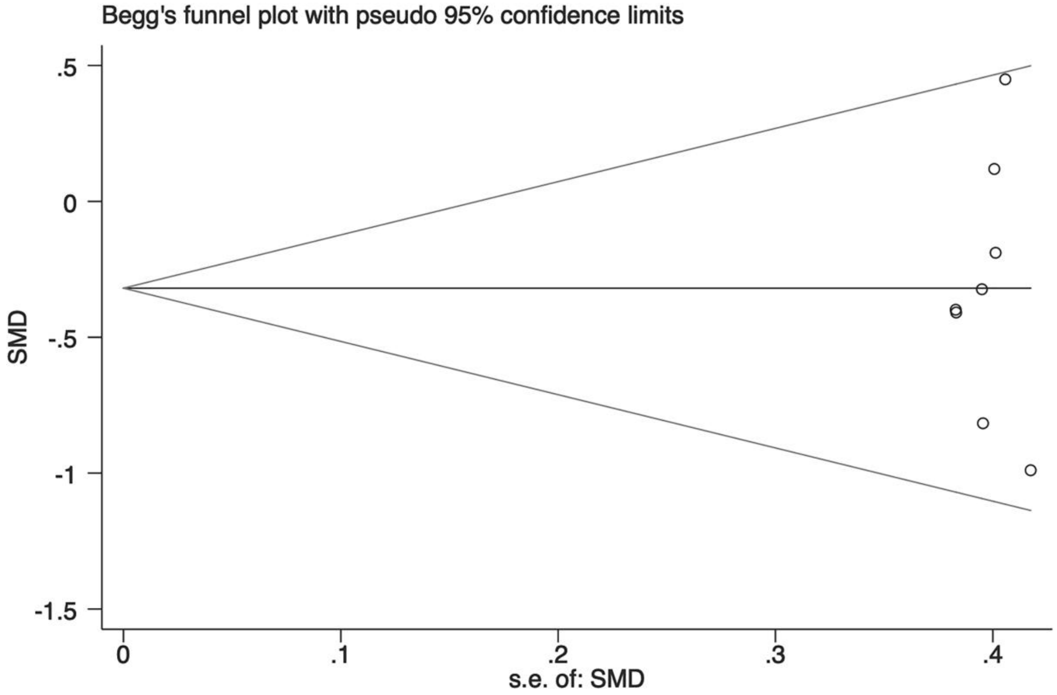

Figure6 Begg test of attention time

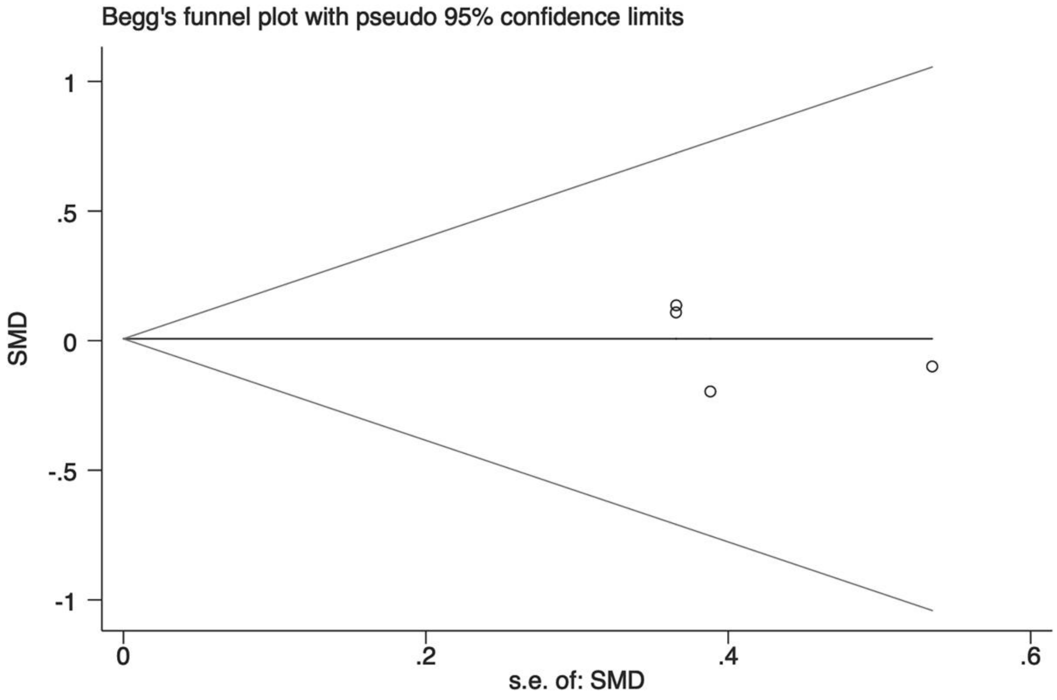

Figure7 Begg test of processing speed

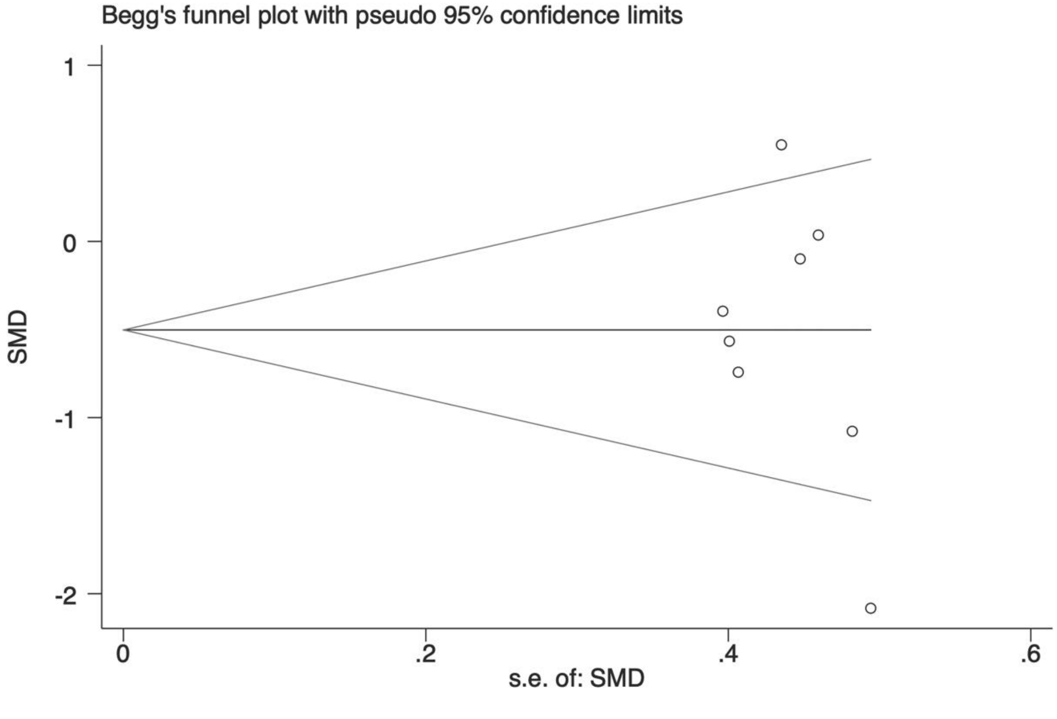

Figure8 Begg test of processing speed time
