# Supplementary material for: The effects of creatine supplementation on cognitive function in adults: a systematic review and meta-analysis
Source: Front Nutr. 2024 Jul 12;11:1424972. doi: 10.3389/fnut.2024.1424972 (PMC11275561; doi:10.3389/fnut.2024.1424972)
Supplement: Supplementary file 2 [file Table_1.DOCX]

**GRADE Assessment**

1.Memory Function

Risk of Bias: Due to the majority of studies employing randomized controlled trial designs and having detailed risk of bias assessments, the risk of bias is considered low.

Consistency: The results show consistent improvements in memory function (SMD = 0.31, 95% CI: 0.17 to 0.44, I² = 23%), with low heterogeneity.

Directness: The studies directly measured memory function, indicating high directness.

Precision: The confidence intervals are narrow, indicating precise results.

Publication Bias: Egger's test results show no significant publication bias.

Conclusion: The certainty of the evidence for memory function is moderate.

2. Processing Speed

Risk of Bias: The studies are well-designed, but the small sample sizes may introduce some risk of bias.

Consistency: The results exhibit high heterogeneity (I² = 63%), indicating poor consistency.

Directness: The studies directly measured processing speed, indicating high directness.

Precision: The confidence intervals are wide, indicating low precision.

Publication Bias: Egger's test results show no significant publication bias.

Conclusion: The certainty of the evidence for processing speed is low.

3. Overall Cognitive Function

Risk of Bias: Most studies are reasonably designed, but there is some risk of bias.

Consistency: The results exhibit high heterogeneity (I² = 75%), indicating poor consistency.

Directness: The studies directly measured overall cognitive function, indicating high directness.

Precision: The confidence intervals are wide, indicating low precision.

Publication Bias: Egger's test results show no significant publication bias.

Conclusion: The certainty of the evidence for overall cognitive function is low.

4. Executive Function

Risk of Bias: The studies are well-designed, but the small sample sizes may introduce some risk of bias.

Consistency: The results exhibit low heterogeneity (I² = 0%), indicating good consistency.

Directness: The studies directly measured executive function, indicating high directness.

Precision: The confidence intervals are wide, indicating low precision.

Publication Bias: Egger's test results show no significant publication bias.

Conclusion: The certainty of the evidence for executive function is low.

5. Attention

Risk of Bias: The studies are well-designed, but the small sample sizes may introduce some risk of bias.

Consistency: The results exhibit high heterogeneity (I² = 61%), indicating poor consistency.

Directness: The studies directly measured attention, indicating high directness.

Precision: The confidence intervals are wide, indicating low precision.

Publication Bias: Egger's test results show no significant publication bias.

Conclusion: The certainty of the evidence for attention is low.
